# Effect of Hydrocortisone vs Pasireotide on Pancreatic Surgery Complications in Patients With High Risk of Pancreatic Fistula: A Randomized Clinical Trial

**DOI:** 10.1001/jamasurg.2019.6019

**Published:** 2020-02-05

**Authors:** Timo Tarvainen, Jukka Sirén, Arto Kokkola, Ville Sallinen

**Affiliations:** 1 Department of Gastroenterological Surgery, University of Helsinki and Helsinki University Hospital, Helsinki, Finland; 2 Department of Transplantation and Liver Surgery, University of Helsinki and Helsinki University Hospital, Helsinki, Finland

## Abstract

**Importance:**

Both hydrocortisone and pasireotide have been shown in randomized clinical trials to be effective in reducing postoperative complications of pancreatic surgery, but to date no randomized clinical trial has evaluated the effectiveness of pasireotide compared with hydrocortisone.

**Objective:**

To assess the noninferiority of hydrocortisone compared with pasireotide in reducing complications after partial pancreatectomy.

**Design, Setting, and Participants:**

A noninferiority, parallel-group, individually randomized clinical trial was conducted at a single academic center between May 19, 2016, and December 17, 2018. Outcome collectors and analyzers were blinded. A total of 281 patients undergoing partial pancreatectomy were assessed for inclusion. Patients younger than 18 years, those allergic to hydrocortisone or pasireotide, patients undergoing pancreaticoduodenectomy with hard pancreas or dilated pancreatic duct, and patients not eventually undergoing partial pancreatectomy were excluded. Modified intention-to-treat analysis was used in determination of the results.

**Interventions:**

Treatment included pasireotide, 900 μg, subcutaneously twice a day for 7 days or hydrocortisone, 100 mg, intravenously 3 times a day for 3 days.

**Main Outcomes and Measures:**

The primary outcome was the Comprehensive Complication Index (CCI) score within 30 days. The noninferiority limit was set to 9 CCI points.

**Results:**

Of the 281 patients (mean [SD] age, 63.8 years) assessed for eligibility, 168 patients (mean [SD] age, 63.6 years) were randomized and 126 were included in the modified intention-to-treat analyses. Sixty-three patients received pasireotide (35 men [56%]; median [interquartile range] age, 64 [56-70] years) and 63 patients received hydrocortisone (25 men [40%]; median [interquartile range] age, 67 [56-73] years). The mean (SD) CCI score was 23.94 (17.06) in the pasireotide group and 30.11 (20.47) in the hydrocortisone group (mean difference, –6.16; 2-sided 90% CI, –11.73 to –0.60), indicating that hydrocortisone was not noninferior. Postoperative pancreatic fistula was detected in 34 patients (54%) in the pasireotide group and 39 patients (62%) in the hydrocortisone group (odds ratio, 1.39; 95% CI, 0.68-2.82; *P* = .37). One patient in the pasireotide group and 2 patients in the hydrocortisone group died within 30 days. In subgroup analyses of patients undergoing distal pancreatectomy, the CCI score was a mean of 10.3 points lower (mean [SD], 16.03 [11.94] vs 26.28 [21.76]; 2-sided 95% CI, −19.34 to −2.12; *P* = .03) and postoperative pancreatic fistula rate was lower (37% vs 67%; *P* = .02) in the pasireotide group compared with the hydrocortisone group.

**Conclusions and Relevance:**

In this study, hydrocortisone was not noninferior compared with pasireotide in patients undergoing partial pancreatectomy. Pasireotide may be more effective than hydrocortisone in patients undergoing distal pancreatectomy.

**Trial Registration:**

ClinicalTrials.gov identifier: NCT02775227; EudraCT identifier: 2016-000212-16

## Key Points

**Question:**

Is hydrocortisone noninferior compared with pasireotide in reducing pancreatic surgery complications?

**Findings:**

In this randomized clinical trial that included 126 patients undergoing partial pancreatectomy, the mean Comprehensive Complication Index score (a measurement of overall postoperative morbidity) was –6.16 points lower in patients receiving pasireotide and the lower limit of the 90% CI crossed the prespecified noninferiority margin (–9). In subgroup analyses of patients undergoing distal pancreatectomy, the mean Comprehensive Complication Index score was significantly lower (10.3 points) in the pasireotide vs hydrocortisone group.

**Meaning:**

In this study, hydrocortisone is not noninferior compared with pasireotide, and pasireotide may be more effective in reducing postoperative complications in patients undergoing distal pancreatectomy.

## Teaser

This randomized clinical trial evaluates the use of pasireotide vs hydrocortisone to reduce postoperative complications in patients undergoing pancreatic surgery.

## Introduction

Pancreatic surgery carries a high risk for complications, and pancreatic fistula remains the main cause of most of the serious complications. Several methods have been introduced during the past decades to address the problem and reduce the incidence of pancreatic fistula and overall complication burden after pancreatic surgery. For pancreaticoduodenectomy, these methods include different types of pancreaticoenteric anastomoses, different drainage strategies, and various stenting methods of the pancreatic duct.^1,2,3,4^ For distal pancreatectomies, different cutting methods and pancreatic stump sealing strategies have been used.^5,6^ Somatostatin analogs, especially octreotide, have also been used for both pancreaticoduodenectomy and distal pancreatectomy. Although the use of octreotide is widespread in some countries, octreotide has not gained full acceptance globally, as meta-analyses have not shown consistent benefit.^7,8^ Despite rigorous research, postoperative pancreatic fistula remains a significant challenge: up to every third patient at high risk will develop a clinically significant pancreatic fistula.^6,9^

Pasireotide is a somatostatin analog with high affinity to 4 of the 5 somatostatin receptors, while octreotide binds only to 2 somatostatin receptors with high affinity.^10^ On this theoretical basis, it can be hypothesized that pasireotide could be more effective in preventing postoperative pancreatic fistula. Clinical proof of this efficacy has been obtained from 1 randomized clinical trial in which pasireotide halved the rate of pancreatic fistula in patients undergoing pancreaticoduodenectomy or distal pancreatectomy.^11^ Although the association between pasireotide and reductions in the incidence of pancreatic fistula has been challenged by several nonrandomized studies or retrospective series,^12,13,14^ to our knowledge, no other prospective randomized clinical trial evaluating the effectiveness of pasireotide exists.

Another pharmacologic strategy to mitigate the rate of postoperative pancreatic fistula is perioperative hydrocortisone treatment. Randomized clinical trials compared hydrocortisone with placebo in patients at high risk for pancreatic fistula undergoing pancreaticoduodenectomy or distal pancreatectomy and showed that hydrocortisone reduces major complications after pancreaticoduodenectomy and the rate of postoperative pancreatic fistula after distal pancreatectomy.^15,16^ Although the study cohorts of these 2 randomized clinical trials were not comparable, hydrocortisone seemed to be similarly effective in reducing pancreatic surgery complications compared with pasireotide.

To evaluate the best pharmacologic therapy to reduce postoperative complications for patients undergoing partial pancreatectomy, we conducted the Hydrocortisone vs Pasireotide in Reducing Pancreatic Surgery Complications (HYPAR) trial. Because pasireotide is substantially more expensive than hydrocortisone and earlier trials suggested equal effectiveness, we aimed to show noninferiority of hydrocortisone in terms of overall postoperative complications in patients undergoing partial pancreatectomy with high risk of pancreatic fistula. Patients undergoing pancreaticoduodenectomy with a hard pancreas or dilated pancreatic duct were not included in the trial because their risk for pancreatic fistula is low.^17^

## Methods

### Study Design and Participants

The HYPAR trial was a single-center, prospective, parallel-group, randomized, noninferiority trial comparing perioperative hydrocortisone with pasireotide in patients at high risk for pancreatic fistula and postoperative complications. The trial was carried out in Helsinki University Hospital, Helsinki, Finland, which is an academic teaching hospital and functions both as a secondary (served population, 1.2 million) and tertiary referral (served population, 1.9 million) center.

The trial was approved by the Finnish National Committee on Medical Research Ethics, Finnish Medicines Agency, Helsinki University Hospital’s ethical committee, and the Helsinki University Hospital Institutional Review Board. All randomized patients gave written informed consent; no financial compensation was provided. This study followed the Consolidated Standards of Reporting Trials (CONSORT) reporting guideline for randomized clinical trials. The protocol is available in Supplement 1.

Patients scheduled for elective partial pancreatectomy (pancreaticoduodenectomy, distal pancreatectomy, enucleation, or other resection) were eligible for inclusion. Exclusion criteria were atrophic pancreas or dilated pancreatic duct (diameter of ≥4 mm) at the resection line in preoperative imaging in patients undergoing pancreaticoduodenectomy, planned total pancreatectomy, allergy or other contraindication for cortisone or pasireotide, age younger than 18 years, or no written informed consent given. Randomization took place before surgery, because the first dose of either hydrocortisone or pasireotide was given before the patient was moved to the operating room. For this reason, additional exclusion criteria were set and checked intraoperatively. Patients were excluded from the trial after randomization if no pancreatic resection took place (eg, disseminated cancer) or total pancreatectomy was performed (ie, no risk of pancreatic fistula). Furthermore, patients undergoing pancreaticoduodenectomy with a hard pancreas or dilated pancreatic duct are at low risk for pancreatic fistula, and thus were excluded from the trial. In other types of resection (enucleation, distal pancreatectomy), patients were not excluded based on their pancreas type during surgery.

### Randomization and Blinding

Patients were randomly allocated 1:1 to receive either perioperative hydrocortisone or pasireotide treatment. The randomization sequence was generated using a computer algorithm with randomly variable block size (2, 4, and 6). The randomization sequence was concealed in opaque and numbered envelopes. The recruiters, treating physician, operating surgeon, researchers, and patients were unaware of the randomization sequence. Patients were randomized by a study nurse by opening the sealed envelope containing the allocation group. The allocated group was concealed from treating physicians, surgeons, researchers, outcome assessors, data collectors, and data analysts until all of the data were collected. The groups were then labeled as 1 and 2, and the primary and secondary end points were analyzed without the knowledge of which group was which. Possible incidents of failed blinding were recorded.

### Intervention

Patients allocated to the pasireotide group received pasireotide, 900 μg, twice a day subcutaneously, starting on the morning of the operation and continuing until the evening dose on postoperative day 6 (14 doses) or until discharge if earlier. Patients allocated to the hydrocortisone group received hydrocortisone, 100 mg, intravenously 3 times a day, starting on the morning of the operation and continuing until the evening dose on postoperative day 2 (9 doses). Patients in both groups were otherwise treated similarly according to the Enhanced Recovery After Surgery protocol.^18^ For pancreaticoduodenectomy, pancreaticojejunostomy was formed in duct-to-mucosa fashion with 4-0 or 5-0 polydioxanone sutures in 2 layers, and 2 closed passive drains were inserted in the abdominal cavity next to the hepaticojejunostomy and pancreaticojejunostomy. All pancreaticoduodenectomies were performed open. For distal pancreatectomy, the pancreas was divided using a linear stapler or, in cases of thick pancreas, the division was performed using a cold knife and the resection line was sutured. Distal pancreatectomies were performed open, laparoscopic, or robot-assisted. One closed passive drain was left next to the pancreas stump after distal pancreatectomy. For both pancreaticoduodenectomies and distal pancreatectomies, the drains were left in place for at least up to postoperative day 3. Amylase levels were measured from the drain output and, if the levels were high, the drains were left in place longer than usual.

### Outcomes

The primary outcome of the trial was the Comprehensive Complication Index (CCI) score within 30 days after the operation. The CCI takes into account all cumulative complications and receives values between 0 and 100. A 10-point difference reflects a 1-grade difference in the established Clavien-Dindo classification.^19^ Secondary outcomes included complications graded by Clavien-Dindo classification, postoperative pancreatic fistula rate (International Study Group of Pancreatic Surgery [ISGPS] classification), postoperative delayed gastric emptying rate (ISGPS classification), postpancreatectomy hemorrhage (ISGPS classification), and readmissions, all within 30 days after the operation, and length of hospital stay assessed at discharge.^20,21,22,23,24^ Outcome measures were assessed during the hospital stay and at outpatient clinic visits or by phone call 30 days after surgery. Other outcome measures included rate of adjuvant therapy within 6 months after surgery in patients with histologically verified cancer. Overall, disease-specific and disease-free survival will be assessed and reported when 5- and 10-year follow-up data are available.

### Statistical Analysis

We chose the CCI as the primary outcome because it is based on the established Clavien-Dindo classification system but takes into account all cumulative complications and is thus more sensitive indicator of overall complication burden. We set the noninferiority limit at 9 points, because a 10-point difference reflects 1 Clavien-Dindo grade difference in complication burden.^19^ We aimed to show that the CCI score in the hydrocortisone group would not be more than 9 points higher than the score in the pasireotide group. Based on published data, we assumed the SD to be 20.^19^ We calculated that 124 patients were needed to show noninferiority of hydrocortisone with 80% power and a 1-sided α level of .05.

Noninferiority was tested by the lower limit of the 90% CI of the mean difference (equivalent of the 95% CI of a 1-sided test). If the lower limit of the CCI mean difference 90% CI is lower than –9, noninferiority is not met. The primary CCI and secondary length of stay outcomes are presented as mean (SD) and compared between groups using independent *t* tests with bootstrapping. Only the primary outcome was assessed using noninferiority testing; the secondary outcomes were assessed using the superiority approach. Categorical secondary outcome measures were compared using a χ^2^ test. Effect sizes are reported either as mean difference with 95% CI or odd ratios (ORs) with 95% CI. All *P* values reported are 2-sided and, for superiority testing, *P* < .05 was considered statistically significant. Data were reported as missing, if necessary, in the tables or in text for each variable. Missing data were omitted from analyses of the particular variable in question and no missing data were imputed. All outcomes were analyzed using modified intention-to-treat analyses, where all randomized patients in whom the study drug was continued after surgery were included in the analyses. Prespecified subgroup analyses were performed using superiority testing for patients undergoing pancreaticoduodenectomy or distal pancreatectomy. Safety interim analysis was planned when 62 patients were randomized.

## Results

A total 281 patients were assessed for eligibility, of whom 168 patients were randomized to either perioperative hydrocortisone or pasireotide treatment between May 19, 2016, and December 17, 2018 (Figure). At surgery, a further 42 patients were excluded from the study, 25 because no pancreatic resection took place, 14 because the pancreas was deemed to be low risk (hard pancreas or dilated pancreatic duct), 1 because of concomitant use of somatostatin analogs, and 2 because of logistic reasons. A total of 126 patients were included in the modified intention-to-treat analyses (Figure). Baseline and operative characteristics were similar between the groups (Table 1 and Table 2). In the whole study cohort the median age of the patients was 66 years, 63 patients (50%) were American Society of Anesthesiologists class 3 or 4, and median Charlson Comorbidity Index score was 2. In the pasireotide vs hydrocortisone group, 35 patients (56%) vs 25 patients (40%) were men, median (interquartile range) age was 64 (56-70) vs 67 (56-73) years, mean (SD) body mass index (calculated as weight in kilograms divided by height in meters squared) was 27.3 (3.6) vs 26.8 (4.3), 31 (49%) vs 32 (51%) patients were American Association of Anesthesiologists class 3 or 4, and preoperative biliary drainage was performed in 21 (33%) vs 15 (24%) patients. Only 2 patients per group underwent neoadjuvant therapy. Fistula risk scores^25^ indicated that patients undergoing pancreaticoduodenectomy in the trial had, on average, intermediate risk for postoperative pancreatic fistula (Table 2). Blinding was reported to have failed in 4 patients (1 unintentionally in both groups with the drug identified for treating physicians, 1 deliberately in the hydrocortisone group by the treating physicians who stopped the drug because of severe nausea, and 1 unintentionally in the hydrocortisone group with the drug identified for data collectors). The drug was prematurely stopped in 7 patients (6 patients in the pasireotide group: 5 for nausea, 1 for suspected allergic reaction at a median of 2 days after the operation, and 1 patient in the hydrocortisone group on the second postoperative day for nausea). For distal pancreatectomy, the pancreatic stump was closed in the pasireotide vs hydrocortisone groups with a stapler in 14 (47%) vs 19 (63%) patients, enforced stapler in 14 (47%) vs 11 (37%) patients, and suturing in 2 (7%) vs 0 patients.

**Figure.**
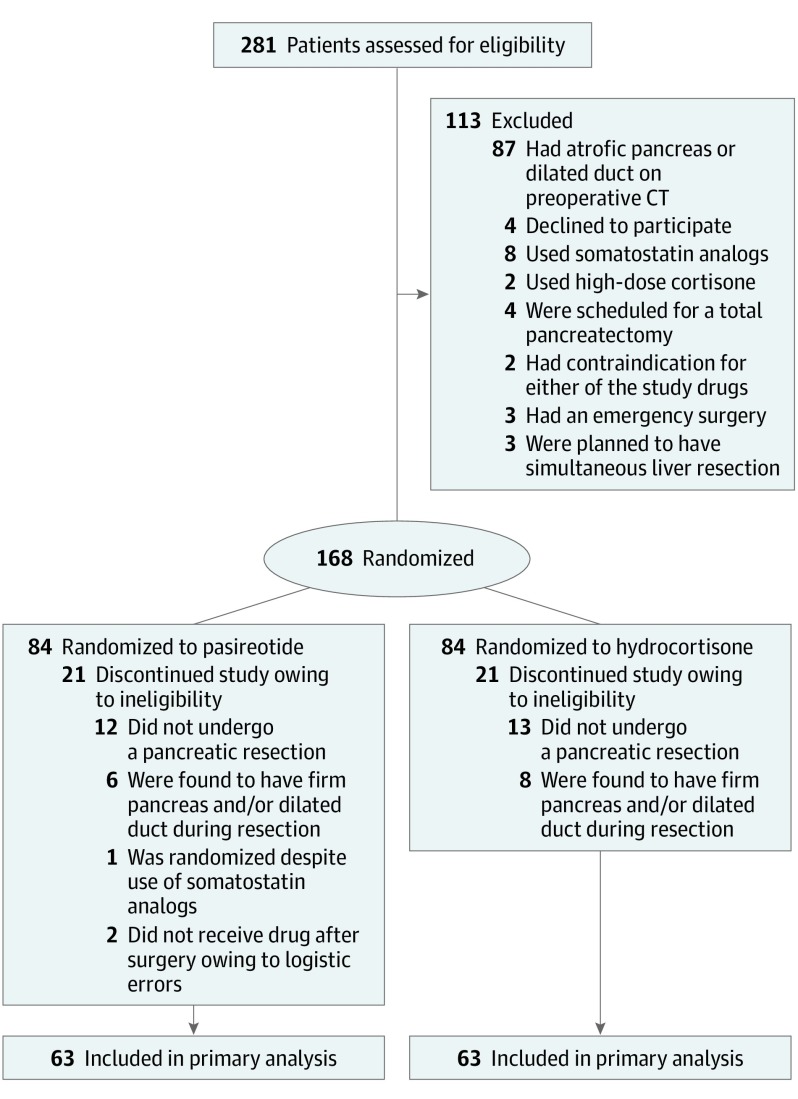
Flow Diagram of Patient Recruitment and Randomization CT indicates computed tomography.

**Table 1.** Baseline Characteristics^a^

Characteristic	No. (%)	
	Pasireotide (n = 63)	Hydrocortisone (n = 63)
Age, median (IQR), y	64 (56-70)	67 (56-73)
Sex, male	35 (56)	25 (40)
BMI, mean (SD)	27.3 (3.6)	26.8 (4.3)
ASA physical status		
1	2 (3)	2 (3)
2	30 (48)	29 (46)
3	29 (46)	29 (46)
4	2 (3)	3 (5)
Charlson Comorbidity Index diseases		
Coronary disease/myocardial infarction	5 (8)	2 (3)
Congestive heart failure	2 (3)	1 (2)
Hypertension	30 (48)	26 (41)
Peripheral vascular disease	3 (5)	3 (5)
Cerebrovascular disease	1 (2)	2 (3)
Hemiplegia	0	0
Dementia	0	1 (2)
COPD or asthma	12 (19)	9 (14)
Connective tissue disease	2 (3)	4 (6)
Liver disease	1 (2)	0
Mild	1 (2)	0
Moderate or severe	0	0
Diabetes	11 (18)	10 (16)
Without complications	11 (18)	10 (16)
With complications	0	0
Kidney disease, moderate or severe	0	1 (2)
Cancer	42 (67)	48 (76)
Local	40 (64)	43 (68)
Metastatic	2 (3)	5 (8)
Leukemia	0	0
Lymphoma	0	0
No comorbidities	7 (11)	11 (18)
Charlson Comorbidity Index category, No. (%)		
Mild (0-2)	45 (71)	37 (59)
Moderate (3-4)	15 (24)	18 (29)
Severe (≥5)	3 (5)	8 (13)
Charlson Comorbidity Index score, mean (SD)	2.0 (1.4)	2.4 (1.9)

Abbreviations: ASA, American Association of Anesthesiologists; BMI, body mass index (calculated as weight in kilograms divided by height in meters squared); COPD, chronic obstructive pulmonary disease; IQR, interquartile range.

^a^ No significant differences were identified between the treatment groups in any baseline variables.

**Table 2.** Operative Characteristics

Characteristic	No. (%)	
	Pasireotide (n = 63)	Hydrocortisone (n = 63)
Preoperative drainage of biliary tract		
No biliary drainage	42 (67)	48 (76)
ERCP	21 (33)	15 (24)
Time from biliary drainage to operation, median (IQR), d	60 (38-79)	69 (38-88)
Resection type		
Pancreaticoduodenectomy	30 (48)	27 (43)
Distal pancreatectomy	30 (48)	30 (48)
Open distal pancreatectomy	8 (27)	4 (13)
Laparoscopic, distal^a^	16 (53)	22 (73)
Laparoscopic, distal converted^a^	6 (20)	4 (13)
Other^b^	3 (5)	6 (10)
Vascular resection		
Venous	4 (6)	5 (8)
Arterial	0	1 (2)
Blood loss during operation, mean (SD), mL	550 (545)	572 (757)
Pancreatic duct diameter, mean (SD), mm^c^	2.7 (1.3)	2.7 (1.6)
Pathologic findings		
Pancreatic ductal adenocarcinoma	14 (22)	13 (21)
Cholangiocarcinoma	4 (6)	5 (8)
Papilla adenocarcinoma	5 (8)	3 (5)
Duodenal adenocarcinoma	3 (5)	1 (2)
IPMN	5 (8)	7 (11)
MCN	4 (6)	4 (6)
PNET	10 (16)	17 (27)
SPN	4 (6)	0
Serous cystadenoma	1 (2)	3 (5)
Papilla adenoma	2 (3)	1 (2)
Dysplasia, excluding papilla	5 (8)	6 (10)
Metastasis of another carcinoma	2 (3)	2 (3)
Other^d^	4 (6)	1 (2)
Neoadjuvant therapy^e^		
None	28 (93)	23 (92)
Gemcitabine plus cisplatin	2 (7)	0
Chemoradiotherapy	0	1 (4)
Carboplatin plus etoposide	0	1 (4)
Fistula Risk Score,^25^ mean (SD)^c^	6.2 (1.7)	6.4 (1.8)
Modified Fistula Risk Score,^9^ mean (SD)^c^	8.3 (1.1)	7.8 (1.4)
Updated Alternative Fistula Risk Score,^17^ mean (SD)^c^	29.3 (10.9)	27.3 (11.3)

Abbreviations: ERCP, endoscopic retrograde cholangiopancreatography; IPMN, intraductal papillary mucinous neoplasia; IQR, interquartile range; MCN, mucinous cystic neoplasia; PNET, pancreatic neuroendocrine tumor; SPN, solid pseudopapillary neoplasm

^a^ Included robot-assisted procedures.

^b^ Three enucleations in the pasireotide group and 3 enucleations, 2 median pancreatectomies, and 1 transduodenal papillectomy in the hydrocortisone group.

^c^ Only for patients undergoing pancreaticoduodenectomy.

^d^ One pancreatic cystadenocarcinoma, 1 poorly differentiated pancreatic cancer, 1 benign choledochal cyst, and 1 lymphoepithelial cyst in the pasireotide group. One adenosquamous carcinoma of the pancreas in the hydrocortisone group.

^e^ Total number of cancer cases (used as denominator) was 30 in the pasireotide group and 25 in the hydrocortisone group.

The mean (SD) CCI score was 23.94 (17.06) in the pasireotide group and 30.11 (20.47) in the hydrocortisone group (mean difference, –6.16; 2-sided 95% CI, –12.81 to 0.48; 2-sided 90% CI, –11.73 to –0.60). The lower limit of the 90% CI crossed the prespecified –9 noninferiority limit, indicating that hydrocortisone was not noninferior (Table 3). Pancreatic fistula rate and other secondary outcomes were similar between the groups, except that postpancreatectomy hemorrhage was more frequent in the hydrocortisone vs pasireotide group (7 [11%] vs 0; *P* = .01) (Table 3). Details of other complications are reported in eTable 1 in Supplement 2. Patients in the pasireotide group had higher glucose levels and required more insulin and more antinausea medication during their hospital stay than patients in the hydrocortisone group (Table 4). One patient in the pasireotide group and 2 patients in the hydrocortisone group died within 30 days after the operation. The patient in the pasireotide group was a 79-year-old man with American Society of Anesthesiologists class 3 and Charlson Comorbidity Index score of 2. He underwent a pancreaticoduodenectomy for a duodenal carcinoma. He had an ISGPS grade C postoperative pancreatic fistula (ie, requires reoperation), which resulted in septic shock and relaparotomy on postoperative day 14. The pancreaticojejunal anastomosis was resutured and drained, but the patient developed multiorgan failure and died on postoperative day 29. Autopsy verified that the cause of death was postoperative pancreatic fistula. The first patient who died in the hydrocortisone group was a 73-year-old man with American Society of Anesthesiologists class 4 and a Charlson Comorbidity Index score of 9. He underwent a pancreaticoduodenectomy for intraductal papillary mucinous neoplasia with worrisome features, indicating risk of malignancy. He had an ISGLS grade C bile leakage leading to a relaparotomy and rehepaticojejunostomy on postoperative day 3. He died of massive aspiration due to delayed gastric emptying on postoperative day 9, which was verified in autopsy. The second patient who died in the hydrocortisone group was a 73-year-old man with American Society of Anesthesiologists class 3 and Charlson Comorbidity Index score of 3. He underwent a distal pancreatectomy for pancreatic cancer. He developed multiorgan failure on postoperative day 6, was transferred to the intensive care unit, and died on postoperative day 7. Autopsy verified that the cause of death was a massive myocardial infarction leading to multiorgan failure.

**Table 3.** Primary and Secondary Outcomes

Outcome	No. (%)		*P* Value^a^	Effect Size, OR (95% CI)
	Pasireotide (n = 63)	Hydrocortisone (n = 63)		
Primary outcome				
Comprehensive Complication index score, mean (SD)	23.94 (17.06)	30.11 (20.47)	.07	–6.16 (–12.81 to 0.48)^b^
Secondary outcome				
Complications, any CD class	54 (86)	60 (95)	.07	3.33 (0.86-12.95)
Clinically significant complications, CD class ≥2	43 (68)	44 (70)	.85	1.08 (0.51-2.29)
Major complications, CD class ≥3	5 (8)	10 (16)	.17	2.19 (0.70-6.82)
Pancreatic fistula				
Any	34 (54)	39 (62)	.37	1.39 (0.68-2.82)
ISGPS class B or C^c^	13 (21)	14 (22)	.83	1.10 (0.47-2. 58)
Delayed gastric emptying				
Any	12 (19)	19 (30)	.15	1.84 (0.80-4.20)
ISGPS class B or C^c^	6 (10)	5 (8)	.75	0.82 (0.24-2.84)
Postoperative hemorrhage				
Any	0	7 (11)	.01	NA^d^
ISGPS class B or C^c^	0	6 (10)	.01	NA^d^
Length of hospital stay, median (IQR), d^e^	8.0 (7.0-13.0)	10 (6.0-13.5)	.95	0.006^f^
Readmission	7 (11)^g^	10 (16)	.46	1.48 (0.53-4.18)
Adjuvant therapy among patients with cancer^h^	20/26 (77)	17/24 (71)	.62	0.73 (0.21-2.59)

Abbreviations: CD, Clavien-Dindo; IQR, interquartile range; ISGPS, International Study Group of Pancreatic Surgery; NA, not applicable; OR, odds ratio.

^a^ *P* values for superiority.

^b^ Mean difference (95% CI). Two-tailed 90% CI was –11.73 to –0.60, which is the equivalent of a 1-sided 95% CI, and the lower limit of this 90% CI was used to test the noninferiority margin.

^c^ ISGPS classification.^21,22,23^

^d^ Effect size could not be calculated owing to a 0 in 1 cell.

^e^ In the pasireotide group, 1 patient died and, in the hydrocortisone group, 2 patients died during the initial hospital stay.

^f^ Effect size was calculated as *r*=*Z*/√N without 95% CI.

^g^ One patient’s data on possible readmissions in his local community hospital were missing.

^h^ Total number of cancer cases was 30 in the pasireotide group and 25 in the hydrocortisone group. Data on possible adjuvant therapy were missing for 4 patients in the pasireotide group and 1 patient in the hydrocortisone group owing to their oncologic consultation at other institutions.

**Table 4.** Postoperative Characteristics

Characteristic	Pasireotide (n = 63)	Hydrocortisone (n = 63)	*P* Value
Postoperative maximum drain amylase, median (IQR), U/L	367 (107-1217)	388 (99-1245)	.85
Postoperative maximum serum glucose, median (IQR), mg/dL	203.6 (180.2-268.5)	182.0 (165.8-223.4)	.02
Total use of rapid- or short-acting insulin during hospital stay, mean (SD), IU^a^	13.0 (28.2)	8.7 (23.8)	.03
Use of antinausea medication during hospital stay, mean (SD), d^a^	4.1 (4.3)	1.9 (4.3)	<.001
Time to oral feeding, median (IQR), d^b^	4 (3-7)	5 (3-9)	>.99

Abbreviation: IQR, interquartile range.

SI unit conversion: To convert serum glucose to millimoles per liter, multiply by 0.0555.

^a^ Total use of insulin and antinausea medication was reported as the mean, although the data were not normally distributed, because the median was 0 in both groups.

^b^ One patient in the pasireotide group and 2 patients in the hydrocortisone group died before proceeding to oral feeding. One patient in the hydrocortisone group had a gastrostomy tube and never proceeded to oral feeding.

In subgroup analysis of patients undergoing pancreaticoduodenectomy, the CCI score was 32.39 in the pasireotide group vs 37.90 in the hydrocortisone group (mean difference, –5.5; 95% CI, −15.46 to 4.12; *P* = .28) (eTable 2 in Supplement 2). In addition, all secondary outcomes were similar between the groups in patients undergoing pancreaticoduodenectomy, except postpancreatectomy hemorrhage, which was more frequent in the hydrocortisone group (4 [15%] vs 0; *P* = .04) (eTable 2 in Supplement 2). Details of complications in patients undergoing pancreaticoduodenectomy are reported in eTable 3 in Supplement 2.

In subgroup analysis for patients undergoing distal pancreaticoduodenectomy, the CCI score for the pasireotide group was, on average, 10.25 less than in the hydrocortisone group (mean, 16.03 vs 26.28; mean difference, –10.25; 95% CI, −19.34 to –2.12; *P* = .03). Eleven patients (37%) in the pasireotide group and 20 patients (67%) in the hydrocortisone group developed a postoperative pancreatic fistula (OR, 3.455; 95% CI, 1.195-9.990; *P* = .02). Four patients (13%) in the pasireotide group and 6 patients (20%) in the hydrocortisone group had a clinically significant (class B or C) postoperative pancreatic fistula (OR, 1.625; 95% CI, 0.408-6.469; *P* = .49) (eTable 4 in Supplement 2). Major complications (Clavien-Dindo 3b or higher) occurred more frequently in the hydrocortisone group than the pasireotide group (6 [20%] vs 0; OR, 0.444 (95% CI, 0.330-0.599; *P* = .02). Details of complications in patients undergoing distal pancreatectomy are reported in eTable 5 in Supplement 2.

## Discussion

The HYPAR trial compared use of perioperative hydrocortisone with pasireotide in patients undergoing partial pancreatectomy and found that hydrocortisone was not noninferior compared with pasireotide in patients undergoing partial pancreatectomy. Both pasireotide and hydrocortisone have been demonstrated to reduce complications of pancreatic surgery,^11,15,16^ but, to our knowledge, they have not been previously compared head-to-head in a randomized clinical trial. Patients in the pasireotide group had, on average, 6.16 fewer CCI points than patients receiving hydrocortisone, but this difference did not reach statistical significance. In subgroup analyses for patients undergoing distal pancreatectomy, pasireotide decreased overall postoperative morbidity significantly and also decreased postoperative pancreatic fistula (pasireotide: 11 patients [37%]; hydrocortisone: 20 patients [67%]), but the study was not powered for subgroup analyses; thus, these findings need to be addressed in another setting for validation.

After the demonstration of superiority of pasireotide reducing postoperative pancreatic fistula compared with placebo in a randomized clinical trial, several retrospective and nonrandomized, prospective cohort studies have been published. These nonrandomized studies were unable to externally verify the benefit of pasireotide in patients undergoing pancreaticoduodenectomy^13^ or partial pancreatectomy.^12,14^ However, because they were not randomized, these studies have various biases, which limit the confidence in their estimates. Cost-effectiveness studies based on the randomized trial suggested that the use of pasireotide reduces the costs of care by approximately $1100 to $1700.^26,27^ However, the cost of 1 dose of pasireotide is $273 in the United States,^26^ which is more than 3-fold the cost in our center (€75 [US $84) and almost 5 times higher than in Germany (€52 [US $58]).^28^

The mechanism of action of pasireotide in reducing pancreatic fistula and, thus, overall complications, is considered to take place via reduction of pancreatic secretion of digestive enzymes and juice. Pasireotide has high affinity to 4 of 5 somatostatin receptors, while the more widely used somatostatin analog octreotide only binds to 2 somatostatin receptor types with high affinity.^10^ Theoretically, pasireotide could be more effective in terms of reducing pancreatic secretion and postoperative pancreatic fistula, but head-to-head randomized clinical trials comparing pasireotide with octreotide are lacking. The mechanism by which hydrocortisone reduces pancreatic surgery complications is thought to be mediated through its anti-inflammatory effects on reducing postoperative pancreatitis and pancreatic fistulas.^15,29^ However, evidence from animal studies suggests that hydrocortisone also, like somatostatin analogs, reduces pancreatic secretion.^30,31^ Given this theoretical background, one must recognize that the exact mechanisms of somatostatin analogs and hydrocortisone in reducing the rates of pancreatic fistula remain unclear.

### Limitations and Strengths

There are limitations in our study. First, this was a single-center trial; a multicenter approach would have given more external validity to our estimates. Second, although we observed fewer complications in the pasireotide group (mean difference, 6.16 CCI points) and demonstrated that hydrocortisone was not noninferior, we were unable to show inferiority of hydrocortisone or superiority of pasireotide. This finding might indicate a type 2 error (ie, false-negative finding owing to a small sample size). However, subgroup analyses suggested that the benefits of pasireotide are more pronounced in patients undergoing distal pancreatectomy. Third, a 3-armed randomized clinical trial with a placebo arm would have provided more strength to this study and a possibility to validate previous findings against placebo. This approach is worthwhile to investigate in a future multicenter setting.

There are also strengths in our trial. First, new treatments should always be compared with the standard or the best treatment available. To our knowledge, our trial is the first to compare 2 pharmacologic postoperative pancreatic fistula prophylaxis agents head-to-head that have earlier been shown to reduce pancreatic surgery complications compared with placebo. Second, we used the CCI as the primary outcome, which is the most sensitive method in classifying the overall complication burden after surgery. Third, patients included in the trial were a median age of 66 years, 50% of them were American Society of Anesthesiologists class 3 or 4, and the median Charlson Comorbidity Index score was 2, indicating that the trial cohort represents the case mix of real-life clinical practice.

## Conclusion

Our results suggest that hydrocortisone is not noninferior compared with pasireotide in reducing complications in patients undergoing partial pancreatectomy. Pasireotide could be more effective in reducing complications in patients undergoing distal pancreatectomy compared with hydrocortisone.

## Notes

Supplement 1. Trial Protocol

Supplement 2. eTable 1. Cumulative Postoperative Complications Within 30 Days eTable 2. Primary and Secondary Outcomes for Patients Undergoing Pancreaticoduodenectomy eTable 3. Cumulative Postoperative Complications Within 30 Days in Pancreaticoduodenectomies eTable 4. Primary and Secondary Outcomes for Patients Undergoing Distal Pancreatectomy eTable 5. Cumulative Postoperative Complications Within 30 Days in Distal Pancreatectomies

Supplement 3. Data Sharing Statement
