# Supplementary material for: Effect of Hydrocortisone vs Pasireotide on Pancreatic Surgery Complications in Patients With High Risk of Pancreatic Fistula: A Randomized Clinical Trial
Source: JAMA Surg. 2020 Feb 5;155(4):291–8. doi: 10.1001/jamasurg.2019.6019 (PMC7042940; doi:10.1001/jamasurg.2019.6019)
Supplement: Supplement 1. — Trial Protocol [file jamasurg-155-291-s001.pdf]

Hydrocortisone vs. pasireotide in preventing pancreatic fistula and other complications after pancreatic resection – a prospective, randomized, controlled trial

Timo Tarvainen, Jukka Sirén, Arto Kokkola, Ville Sallinen

(English translation of the trial protocol originally written in Finnish)

Background

Pancreatic surgery is associated with a significant risk for postoperative complications, particularly for pancreatic anastomotic insufficiency, which lead to pancreatic fistula and other complications. Various methods have been examined in order to reduce this complication. Recently a large randomized controlled trial was published from the United States, which showed that pasireotide is effective in reducing postoperative pancreatic fistula (Allen et al., 2014). Based on this trial, our institution began the use of pasireotide in preventing postoperative pancreatic fistula in high risk patients, whose pancreas is soft and/or pancreatic duct narrow. Last year a Finnish research group published a similar study, which showed that perioperative cortisone treatment reduces complications and postoperative fistulas similarly as pasireotide in the aforementioned trial (Laaninen et al., 2014,2015). In both trials, the experimental drug was compared to placebo, and no study comparing these two drugs to one another has been carried out. Since pasireotide is more expensive than cortisone, it is important to find out whether the effecacy in preventing complications is at the same level, and cortisone could replace pasireotide as the standard preventive drug.

Hypothesis

Cortisone is non-inferior to pasireotide in preventing postoperative pancreatic fistula and complications

Methods

Sites

Abdominal Center, Helsinki University Hospital

Intervention groups

1) Perioperative pasireotide treatment
   Patient receives 900 microg of pasireotide s.c. twice a day starting at the morning of the day of surgery, and continues until the 6th postoperative days evening dose (total 14 doses)
2) Perioperative cortisone treatment
   Patient receives 100mg of hydrocortisone i.v. three times a day starting at the morning of the day of surgery, and continues until the 2th postoperative days evening dose (total 9 doses)

Apart from the drug give according to the intervention group (pasireotide or cortisone), the treatment of patient is according to the normal routine after pancreatic resection.

Study patients

Inclusion criteria

- Patient scheduled for pancreatic resection (pancreaticoduodenectomy, distal resection, enuclation, or other resection)

Exclusion criteria

- Atrophic pancreas or markedly dilated pancreatic duct visible at computed tomography at the site where pancreaticojejunostomy will be reconstruction (applies only to patients scheduled for pancreaticoduodenectomy)
- Patient is scheduled for total pancreatectomy
- Allergy or other contraindication for cortisone or pasireotide
- Age < 18 y
- No written informed consent

Criteria to remove patients from analyses after randomization:

- Pancreatic resection was not performed (e.g. disseminated cancer)
- Hard pancreas and/or dilated pancreatic duct (so called low risk pancreas) (applies only to patients undergoing pancreaticoduodenectomy – the decision is made intraoperatively)
- Patient withdraws consent (data collected until withdrawal will be used in the study)

NOTE! Unmasking or premature stopping of the trial drug will not remove patient from the trial.

Randomization

Patients will be randomized 1:1 to receive either perioperative pasireotide or cortisone treatment. Randomization will be done computer assised using randomly variable block size. All patients, who fullfil inclusion criteria and do not meet exclusion criteria, will be randomized and allocated treatment will be commenced before surgery. A piece of paper indicating the allocated group will be put in an opaque envelope (so called safety envelope). The envelopes will be numbered and they are opened in numerical order.

Blinding

Because one drug is given s.c. and the other i.v., patients and nurses cannot be blinded. The study will be commenced so called single-blided, in which the treating doctors and researches are unaware of the allocation group. The envelope in which the allocation group is concealed will be opened by a study nurse, who marks this in Miranda (EHR) as "pancreatic fistula study drug" instead of the drug's real name. The real name of the drug is marked on a separate place, so that the treating doctors will not see which drug the patient will receive inadvertently. The administration of drugs will be scheduled so that they will occur only after the treating doctors rounds, so that the blinding will not be unmasked. In case the treating doctors need to know which drug was administered (e.g. significant adverse event suspicion, change in treatment, etc), the blinding will be unmasked, and the treating doctor may find out which drug the patient has received. Any unblinding will always be marked in Miranda (EHR) either in orders section or in normal health record text on Surgery page.

Outcome measures

Primary outcome

Comprehensive complication index 30 days postoperatively

Secondary outcoems

1. Complications (Clavien-Dindo classification), 30 days
2. Rate and severity of postoperative pancreatic fistula (ISGPS classification), 30 days
3. Rate and severity of relayed gastric emptying (ISGPS classification), 30 days
4. Rate and severity of post-pancreatectomy haemorrhage (ISGPS classification), 30 days
5. Length of hospital stay, days
6. Readmissions, 30 days
7. Number of patients received adjuvant therapy among patients with cancer diagnosis

Tertiary outcomes

1. Overall survival, 1, 5, 10y
2. Disease-free survival, 1, 5, 10y
3. Disease-specific survival, 1, 5, 10y

Outcome measures will be analyzed in following subgroups: 1) Patients who have undergone pancreaticoduodenectomy, 2) Patients who have undergone distal resection

Follow-up

Patients will be followed according to normal postoperative routine, either at Helsinki University Hospital or in referring hospital. If the follow-up will be in referring hospital, patient records from these hospitals will be requested 1, 5 and 10 years from surgery. There is no extra outpatient clinic visits outside normal routine follow-up.

Expenses

At the moment, pasireotide is routinely used in patient eligibile for the trial in Helsinki University Hospital. Patients will be randomized 1:1 to receive either pasireotide or cortisone. Cortisone is cheaper that pasireotide, so the study will not generate extra expenses, but on the contrary it will save expenses. Hydrocortisone (Solucortef) 100mg injection costs 5.97EUR, so the drug expenses in this arm are total 53,73EUR. Pasireotidi (Signifor) 900microg injection costs 73,68EUR, so the drug expenses in this arm are total 1031.52EUR.

Collected parameters

See appendix 1.

Sample size

Sample size is calculated based on primary outcome, Comprehensive complication index (CCI). CCI is based on Clavien-Dindo (C-D) classification, and CCI takes into account cumulative burden of complications better than C-D classification (Slankamenac et al., 2014). Thus CCI is more sensitive and requires smaller sample size compared to C-D classification. Clinically relevant difference in CCI is set at 10 points. This study will aim to show that cortisone-group does not have more than 9 CCI points more than in the pasireotide-group. Standard deviation (SD) is estimated to be 20 based on earlier publications. Based on sample size calculations 62 patients are required per group to show this, thus total 124 patients.

Schedule

The study will be commenced directly after receiving the permissions to conduct it, estimated at spring 2016. Approximately 100 pancreatic resections are being performed in Helsinki University Hospital annually, and approximately 50 – 60 of these are estimated to be suitable for the trial. Recruiting period is estimated to take approximately 2 years, after which primary and secondary outcome measures can be analyzed and published. Tertiary outcomes will be analyzed when 1, 5, or 10 years have passed from the recruitment of the last patient. Interrim analysis will be made when half of the patients have been recruiting, meaning at 62 patients. Complication outcomes will be analyzed, and based on these the decision whether it is safe to continue study will be made.

Statistical analysis plan

Non-inferiority will be tested by lower limit the mean difference's 90% confidence interval (equivalent of 95% confidence interval of one-sided test). If the lower limit of CCI's mean difference's 90% confidence interval is under -9, non-inferiority is not met. Of primary and secondary outcomes, CCI and length of stay will be presented as mean (standard deviation) and compared between groups using independent t-test with bootstrapping. Only primary outcome will be assessed using non-inferiority testing, and secondary outcomes will be assessed using superiority approach. Categorical secondary outcome measures are compared using Chi square test. Effect sizes are reported either as mean difference with 95% confidence interval or odd ratios (OR) with 95% confidence interval. All p-values reported are two-sided and for superiority testing. P-value <0.05 was considered statistically significant. Missing data, if any, will bereported. Missing data are omitted from analyses of the particular variable in question and no missing data is imputed. All outcomes will be analyzed using modified intention-to-treat analyses, where all randomized patients in whom the study drug is continued after surgery are included in the analyses. Subgroup analyses are performed using superiority testing for patients undergoing pancreaticoduodenectomy or distal pancreatectomy.

Registration

The study will be registered in clinicaltrials.gov before recruitment.

Data protection

The collected data and list of patients will be held in locked room and/or electronical data on a computer which is protected with password and encryption.

References

Allen PJ, Gönen M, Brennan MF, Bucknor AA, Robinson LM, Pappas MM, Carlucci KE, D'Angelica MI, DeMatteo RP, Kingham TP, Fong Y, Jarnagin WR. 2014.
Pasireotide for postoperative pancreatic fistula. N Engl J Med 370:2014–2022.

Laaninen M, Sand J, Vasama K, Laukkarinen J. Postoperative complications following pancreaticoduodenectomy (PD) in high-risk patients can be reduced with hydrocortisone treatment. A randomised controlled trial. Pancreatology 2015 15;3:S122

Laaninen M, Sand J, Vasama K, Laukkarinen J. Whipplen leikkauksen jälkeisiä komplikaatioita voidaan vähentää hydrokortisonihoidolla. Randomoitu

plasebokontrolloitu tutkimus. Suomen Kirurgiyhdistys ry 2015 36;4

Slankamenac K, Nederlof N, Pessaux P, de Jonge J, Wijnhoven BPL, Breitenstein S, Oberkofler CE, Graf R, Puhan MA, Clavien P-A. 2014. The Comprehensive Complication Index. Ann Surg 260:757–763.

Collected parameters
Name
Social security number

Date of birth
Gender (1 = male, 2 = female)

Charlson comorbidity index

Medication
- Anticoagulation (0 = no, 1 = yes)
- Immunosuppression (0 = no, 1 = yes)
- Cortisone (0 = no, 1 = yes)

Date of diagnosis of pancreatic tumor (suspicion) (=computed tomography date)

Preoperative diagnosis (1 = pancreatic cancer, 2 = cholangiocarcinoma, 3 = IPMN, 4 = NET, etc)

Neoadjuvant therapy (0 = no, 1 = gemcitabine, 2 = folfirinox, 4 = chemoradiotherapry, 4 = other)

Randomization group (1 = signifor, 2 = cortisone)

Date of surgery

Type of surgery (1 = whipple, 2 = distal resection, 3 = enucleation, 4 = median pancreatectomy, 4 = exploration, 5 = total pancreatectomy, 6 = other)
Venous resection (0 = no, 1 = portal/SMV, tangential or end-to-end, 2 = porta/SMV,
Arterial resection (0 = no, 1 = yes)
Pancreaticojejunostomytype (1 = Heidelberg, 2 = Sirén, 3 = other)
Consistency of pancreas (1 = hard, 2 = soft, normal)
Pancreatic duct at operation (1 = small, normal, 2 = dilated)
Pancreatic duct size, computed tomography, mm
Drains (0 = no, 1 = one drain, 2 = two drains)

Was study drug continued after operation (1 = yes, 2 = no, hard pancreas/dilated duct in patient undergoing whipple, 3 = no, no operation, 4 = no, total pancreatectomy)

Complications, 30 days from operation
- Pancreatic fistula, ISGPS grade
- DGE, ISGPS grade
- Postop haemorrhagia, ISGPS grade

First complication, C-D class
First compication, description (1 = pancreatic fistula, 2 = DGE, 3 = postop haemorrhagia, 4 = pulmonary embolism, 5 = pneumonia, 6 = UTI, 7 = superficial wound infection, 8 = deep wound infection, etcetc)

Second complication, C-D class
Second compication, description (1 = pancreatic fistula, 2 = DGE, 3 = postop haemorrhagia, 4 = pulmonary embolism, 5 = pneumonia, 6 = UTI, 7 = superficial wound infection, 8 = deep wound infection, etcetc)

Third complication, C-D class
Third compication, description (1 = pancreatic fistula, 2 = DGE, 3 = postop haemorrhagia, 4 = pulmonary embolism, 5 = pneumonia, 6 = UTI, 7 = superficial wound infection, 8 = deep wound infection, etcetc)

Fourth complication, C-D class
Fourth compication, description (1 = pancreatic fistula, 2 = DGE, 3 = postop haemorrhagia, 4 = pulmonary embolism, 5 = pneumonia, 6 = UTI, 7 = superficial wound infection, 8 = deep wound infection, etcetc)

Fifth complication, C-D class
Fifth compication, description (1 = pancreatic fistula, 2 = DGE, 3 = postop haemorrhagia, 4 = pulmonary embolism, 5 = pneumonia, 6 = UTI, 7 = superficial wound infection, 8 = deep wound infection, etcetc)

Sixth complication, C-D class
Sixth compication, description (1 = pancreatic fistula, 2 = DGE, 3 = postop haemorrhagia, 4 = pulmonary embolism, 5 = pneumonia, 6 = UTI, 7 = superficial wound infection, 8 = deep wound infection, etcetc)

Postoperative amylase level at day 3
Postoperative drain amylase level at day 3
Postoperative blood sugar level, highest during hospital stay

Date of commencement of normal per oral feeding

Date of discharge
Destination of discharge (1 = home, 2 = primary care ward, 3 = referring hospital)
Referring hospital's name (1 = Helsinki University Hospital, 2 = Lappeenranta, 3 = Lahti, 4 = Kotka, etc)

PAD

Histology (1 = pancreatic ductal adenocarcinoma, 2 = cholangiocarcinoma, 3 = IPMN, 4 = NET, etc)
pT category
pN category

pM category
Number of examined lymph nodes
Number of metastatic lymph nodes
R category

Re-admission, 30 days (0 = ei, 1 = yes)
Reason, free text

Was the blinding unmasked to treating doctors (0 = ei, 1 = yes, inadvertently, 2 = yes, advertently unmasked)
Reason, free text

Date of study drug stopping

Adverse event of study drug (0 = no adverse events, 1 = nausea, 2 = elevated blood sugar, etc)

Postoperative outpatient clinic visit (0 = no, 1 = yes, Helsinki University Hospital)

Adjuvant therapy (0 = no, 1 = gemcitabine, 2 = folfirinox, etcetc)
Date of commencement of adjuvant therapy

Recurrence (0 = no, 1 = local, 2 = liver, 3 = lungs, 4 = peritoneal carcinosis, 5 = multiple)
Date of recurrence

Death (0 = no, 1 = yes, caused by pancreatic tumor, 2 = yes, unrelated to pancreatic tumor, 3 = yes, surgery complication)
Date of death
Last date of follow-up
Remission (0 = no, 1 = yes)

Patient withdrawn consent (0 = no, 1 = yes)
Date of withdrawal

(the study protocol in Finnish)

Hydrocortisone vs. pasireotide in preventing pancreatic fistula and other complications after pancreatic resection - a prospective, randomized, controlled trial

Timo Tarvainen, Jukka Sirén, Arto Kokkola, Ville Sallinen

Vatsakeskus, Helsingin yliopistollinen keskussairaala

Tausta
Haimaleikkauksiin liittyy merkittävä riski komplikaatioihin, erityisesti haimasuolisauman pettämiseen, joka johtaa haimafisteliin ja sitä kautta myös muihin komplikaatioihin. Lukuisia eri menetelmiä on tutkittu tämän komplikaation vähentämiseksi. Hiljattain julkaistiin Yhdysvalloista suuri randomoitu sarja, jossa osoitettiin pasireotidin vaikuttavuus haimafistelien vähentämiseksi (Allen ym., 2014). Tämän tutkimuksen perusteella klinikassamme aloitettiin pasireotidin käyttö haimafistelien vähentämiseksi ns. korkean riskin potilailla, joilla haima on pehmeä ja/tai haimatiehyt on kapea. Viime vuonna kuitenkin suomalainen tutkimusryhmä julkaisi vastaavanlaisen tutkimuksen, jossa osoitettiin perioperatiivisen kortisolihoidon vähentävän komplikaatioita ja fisteleitä kutakuinkin samanverran kuin pasireotidi toisessa julkaisussa (Laaninen ym. 2014, 2015). Molemmissa tutkimuslääkettä verrattiin placeboon, eikä näitä kahta lääkettä vertailevaa tutkimusta ole tehty. Koska pasireotidi on kalliimpi hoito kuin kortisoni, on tärkeää selvittää ovatko lääkkeiden estovaikutus samaa luokkaa, jolloin voitaisiin siirtyä käyttämään kortisonia pasireotidin sijaan.

Hypoteesi
Kortisoni ei ole huonompi kuin pasireotidi haimafistelien ja komplikaatioiden ilmaantumisen vähentämisessä

Menetelmät

Tutkimuspaikat
Vatsakeskus, Helsingin yliopistollinen keskussairaala

Interventioryhmät
1) Perioperatiivinen pasireotidihoito

   Potilas saa pasireotidiä 900 mikrog s.c. kaksi kertaa päivässä alkaen leikkauspäivän aamuna, jatkuen 6. postoperatiivisen päivän ilta-annokseen saakka (yhteensä 14 annosta)

2) Perioperatiivinen kortisonihoito

   Potilas saa hydrokortisonia 100 mg i.v. kolme kertaa päivässä alkaen leikkauspäivän aamuna, jatkuen 2. postoperatiivisen päivän ilta-annokseen saakka (yhteensä 9 annosta)

Lukuunottamatta ryhmämukaista lääkehoitoa (pasireotidi tai kortisoni) potilaiden hoito noudattaa rutiininomaista normaalia haimaleikkauspotilaiden hoitoa.

Tutkimuspotilaat

Inkluusiokriteerit

- Haimaleikkaukseen tuleva potilas (pankreatikoduodenektomia, distaalinen resektio, enukleaatio, tai muu resektio)

Ekskluusiokriteerit

- Tietokonetomografiassa atrofinen haima tai merkittävästi laajentunut haimatiehyt, haiman osassa johon haimasuolisauma tullaan rakentamaan (koskee vain pankreatikoduodenektomiaan tulevia potilaita).
- Potilaalle suunnitellaan totaalipankreatektomiaa
- Allergia tai muu kontraindikaatio kortisonille tai pasireotidille
- Ikä < 18 v
- Ei annettua kirjallista suostumusta

Kriteerit poistaa potilas tutkimuksesta randomisaation jälkeen:

- Haimaresektiota ei tehdäkään (esim. levinnyt syöpä)
- Haima on kova ja/tai haimatiehyt on laaja (ns. pienen riskin haima) (koskee vain potilaita, joille on tehty pankreatikoduodenektomia - päätös tehdään intraoperatiivisesti)
- Potilaalle tehdäänkin totaalipankreatektomia
- Potilas haluaa keskeyttää tutkimuksen (keskeyttämiseen mennessä kerättyjä tietoja käytetään tutkimuksessa).

HUOM! Sokkouttamisen purkaminen ja tutkimuslääkkeen ennenaikainen lopettaminen ei poista potilasta tutkimuksesta.

Satunnaistaminen

Potilaat satunnaistetaan 1:1 saamaan joko perioperatiivinen pasireotidi tai kortisoni hoito. Satunnaistaminen tehdään tietokoneavusteisesti käyttäen vaihtelevaa blokkikokoa. Kaikki potilaat, jotka täyttävät inkluusiokriteerit, eikä ekskluusiokriteerit täyty satunnaistetaan, ja arvotun ryhmän mukainen hoito aloitetaan ennen leikkausta. Randomisaatioryhmän kertova lappu laitetaan kirjekuoreen, josta ei näy läpi (ns. turvakuori). Kirjekuoret numeroidaan, ja ne avataan numerojärjestyksessä.

Sokkoutus

Koska toinen lääke annetaan s.c. ja toinen i.v., ei potilaita tai hoitohenkilökuntaa voida sokkouttaa. Tutkimus tehdään ns. yksöissokkeutetusti, jolloin hoitavat lääkärit ja tutkijat eivät tiedä kumpaan ryhmään potilas kuuluu. Potilaan ryhmän kertovan kirjekuoren avaa potilasta hoitava hoitaja, joka kirjaa Mirandan lääkelistalle lääkkeen nimeltä "haimafistelitutkimuslääke", ja sen lisätietoihin tarkemmin kumpi lääke on kyseessä. Näin ollen lääkärit eivät tiedä kumpaa lääkettä potilas tulee saamaan. Lääkkeiden annot ajoitetaan niin, että ne tapahtuvat vasta lääkärin kierron jälkeen, jotta annettava lääke ei paljastu lääkäreille. Mikäli hoitavilla lääkäreillä on tarve tietää kumpaa lääkettä potilas on saanut (esim. merkittävä haittavaikutusepäily, hoidon muutos, tms), puretaan sokkoutus, ja

lääkäri voi käydä katsomassa kumpaa lääkettä potilas on saanut. Sokkoutuksen purkamisesta/purkautumisesta tehdään aina merkintä Mirandan määräysosioon (ns. nuijan alle) tai sairaskertomustekstiin KIR-lehdelle.

Tutkittavat muuttujat

Päävastemuuttuja

Comprehensive complication index 30 vrk postoperatiivisesti

Sekundaariset vastemuuttujat

1. Komplikaatiot (Clavien-Dindo luokitus), 30 vrk
2. Haimafistelien esiintyvyys ja vaikeusaste (ISGPS luokitus), 30vrk
3. Delayed gastric emptying esiintyvyys ja vaikeusaste (ISGPS luokitus), 30vrk
4. Post-pancreatectomy hemorrhage esiintyvyys ja vaikeusaste (ISGPS luokitus), 30vrk
5. Sairaalassaoloaika, päiviä
6. Readmissiot, 30vrk
7. Adjuvanttihoidon aloittaneiden potilaiden lkm/syöpäpotilaiden lkm

Tertiääriset vastemuuttujat

1. Overall survival, 1, 5, 10v
2. Disease-free survival, 1,5, 10v
3. Disease-specific survival, 1, 5, 10v

Vastemuuttujat analysoidaan myös alaryhmittäin: 1) Potilaat, joille tehty pankreatikoduodenektomia, 2) Potilaat, joille tehty distaalinen haimaresektio.

Seuranta

Potilaita seurataan normaalin rutiiniprotokollan mukaisesti, joko HYKS:ssä tai lähettävässä sairaalassa. Mikäli seuranta tapahtuu muussa sairaalassa, pyydetään näistä sairaaloista sairaskertomustekstit 1, 5, ja 10v leikkauksesta. Tutkimukseen ei kuulu ylimääräisiä kontrollikäyntejä.

Kustannukset

Tutkimukseen otettaville potilaille on tällä hetkellä HYKS:ssä rutiinihoitona perioperatiivinen pasireotidihoito. Tutkimuksessa randomoidaan potilaan 1:1 joko pasireotidihoitoon tai kortisonihoitoon. Kortisoni on halvempaa kuin pasireotidi, joten tutkimus ei aiheuta lisäkustannuksia, vaan päinvastoin vähentää kustannuksia. Hydrokortisoni (Solucortef) 100mg injektio maksaa 5,97 euroa, joten tämän haaran lääkekustannukset ovat yhteensä 53,73 euroa. Pasireotidi (Signifor) 900mikrog injektio maksaa 73,68 euroa, joten tämän haaran lääkekustannukset ovat yhteensä 1031,52 euroa.

Kerättävät parametrit

Katso Liite 1.

Otoskoko

Otoskoko lasketaan päävastemuuttujan eli Comprehensive complication index:n (CCI) perusteella. CCI on Clavien-Dindo (C-D) komplikaatioluokitteluun perustuva luokitus, joka ottaa C-D luokittelua paremmin huomioon sen, että samalla potilaalla voi esiintyä useampi

komplikaatio (Slankamenac ym., 2014). Näin ollen CCI on herkempi, ja vaatii pienemmän otoskoon, kuin jos käytettäisiin C-D luokittelua. Kliinisesti merkittävä CCI ero asetetaan 10 pisteeseen. Tutkimuksella pyritään osoittamaan, ettei kortisoni-ryhmässä CCI ole yli 9 pistettä enemmän kuin pasireotidiryhmässä. Standardideviaatio (SD) ajatellaan aikaisempien julkaisujen perusteella olevan 20. Otoskokolaskelmien tämän osoittamiseksi vaaditaan 62 potilasta per ryhmä, eli yhteensä 124 potilasta.

Aikataulu

Tutkimus aloitetaan heti tutkimuslupien saamisen jälkeen, arviolta keväällä 2016. Haimaleikkauksia tehdään HYKS:ssä noin 100 kpl / vuosi, ja näistä arvioidaan n. 50-60 olevan sopivia tutkimukseen. Rekrytointivaiheen arvioidaan kestävän n. 2 vuotta, jonka jälkeen voidaan analysoida ja julkaista tulokset primaari- ja sekundaarivastemuuttujien osalta. Tertiäärimuuttujien osalta tulokset analysoidaan 1, 5 ja 10 vuotta viimeisen potilaan rekrytoimisesta. Tutkimukssa tehdään välianalyysi puolessa välissä, eli kun 62 potilasta on rekrytoitu. Tulokset arvoidaan komplikaatioiden osalta, joiden perusteella päätetään onko tutkimusta turvallista jatkaa.

Rekisteröinti

Tutkimus rekisteröidään ennen potilaiden rekrytoimista clinicaltrials.gov-palveluun.

Tietosuoja

Kerättävä aineisto ja potilaslistat säilytetään lukitussa tilassa ja/tai sähköiset tiedot tietokoneilla salasanalla ja suojauksella suojattuina.

Viitteet

Allen PJ, Gönen M, Brennan MF, Bucknor AA, Robinson LM, Pappas MM, Carlucci KE, D'Angelica MI, DeMatteo RP, Kingham TP, Fong Y, Jarnagin WR. 2014. Pasireotide for postoperative pancreatic fistula. N Engl J Med 370:2014–2022.

Laaninen M, Sand J, Vasama K, Laukkarinen J. Postoperative complications following pancreaticoduodenectomy (PD) in high-risk patients can be reduced with hydrocortisone treatment. A randomised controlled trial. Pancreatology 2015 15;3:S122

Laaninen M, Sand J, Vasama K, Laukkarinen J. Whipplen leikkauksen jälkeisiä komplikaatioita voidaan vähentää hydrokortisonihoidolla. Randomoitu plasebokontrolloitu tutkimus. Suomen Kirurgiyhdistys ry 2015 36;4

Slankamenac K, Nederlof N, Pessaux P, de Jonge J, Wijnhoven BPL, Breitenstein S, Oberkofler CE, Graf R, Puhan MA, Clavien P-A. 2014. The Comprehensive Complication Index. Ann Surg 260:757–763.

Kerättävät parametrit

Nimi
Sotu

Syntymäaika
Sukupuoli (1=mies, 2=nainen)

Charlson comorbidity index

Lääkitykset

Antikoagulaatio (0 = ei, 1 = kyllä)
Immunosuppresseio (0 = ei, 1 = kyllä)
Kortisoni (0 = ei, 1 = kyllä)

Haimatuumori(epäilyn) diagnoosipvm (=TT-kuvaus)

Preop diagnoosi (1 = haimasyöpä, 2 = kolangioca, 3 = IPMN, 4 = NET, jne)

Neoadjuvanttihoito (0 = ei, 1=gemsitabiini, 2=folfirinox, 3=kemosäde, 4=muu)

Randomisaatioryhmä (1 = signifor, 2 = kortisoni)

Leikkauspvm

Leikkaustyyppi (1 = whipple, 2 = distaalinen resektio, 3 = enukleaatio, 4 = mediaani pankreatektomia, 4 = eksploraatio, 5 = totaalipankreatektomia, 6 = muu)
Venaresektio (0 = ei, 1 = porta/VSM, tangentiaali tai pää-päätävasten, 2 = porta/VSM,
Arteriaresektio (0 = ei, 1 = kyllä)
Pankreatikojejunostomiatyyppi (1 = Heidelberg, 2 = Sirén, 3 = muu)
Haiman konsistenssi (1 = kova, 2 = pehmeä, normaali)
Haimatiehyen koko leikkauksessa (1 = pieni, normaali, 2 = laajentunut)
Haimatiehyen koko, TT kuva, mm
Dreenit (0 = ei, 1 = yksi dreeni, 2 = kaksi dreeniä)

Jatkettiinko tutkimuslääkettä (1=kyllä, 2=ei, kova haima/iso tiehyt whipple potilaalla, 3=ei, ei leikkausta, 4=ei, totaalipankreatektomia)

Komplikaatiot, 30vrk leikkauksesta

Haimafisteli, ISGPS grade
DGE, ISGPS grade
Postop hemorrhagia, ISGPS grade

1. komplikaatio, C-D luokka
1. komplikaatio, kuvaus (1 = haimafisteli, 2 = DGE, 3 = postop vuoto, 4 = keuhkoembolia, 5 = pneumonia, 6 = VTI, 7 = pinnallinen haavainfektio, 8 = syvä haavainfektio, jnejne)

2. komplikaatio, C-D luokka
2. komplikaatio, kuvaus (1 = haimafisteli, 2 = DGE, 3 = postop vuoto, 4 = keuhkoembolia, 5 = pneumonia, 6 = VTI, 7 = pinnallinen haavainfektio, 8 = syvä haavainfektio, jnejne)

3. komplikaatio, C-D luokka
3. komplikaatio, kuvaus (1 = haimafisteli, 2 = DGE, 3 = postop vuoto, 4 = keuhkoembolia, 5 = pneumonia, 6 = VTI, 7 = pinnallinen haavainfektio, 8 = syvä haavainfektio, jnejne)

4. komplikaatio, C-D luokka
4. komplikaatio, kuvaus (1 = haimafisteli, 2 = DGE, 3 = postop vuoto, 4 = keuhkoembolia, 5 = pneumonia, 6 = VTI, 7 = pinnallinen haavainfektio, 8 = syvä haavainfektio, jnejne)

5. komplikaatio, C-D luokka
5. komplikaatio, kuvaus (1 = haimafisteli, 2 = DGE, 3 = postop vuoto, 4 = keuhkoembolia, 5 = pneumonia, 6 = VTI, 7 = pinnallinen haavainfektio, 8 = syvä haavainfektio, jnejne)

6. komplikaatio, C-D luokka
6. komplikaatio, kuvaus (1 = haimafisteli, 2 = DGE, 3 = postop vuoto, 4 = keuhkoembolia, 5 = pneumonia, 6 = VTI, 7 = pinnallinen haavainfektio, 8 = syvä haavainfektio, jnejne)

Postoper amylaasio 3vrk:na, pitoisuus
Postoper dreeni-amylaasi 3vrk, pitoisuus
Postoper verensokeri, suurin pitoisuus hoitojaksolla

Milloin aloittanut tavallisen ruuan, pvm

Kotiutumispäivä, pvm
Kotiutuminen (1=kotiin, 2=TK vuodeos, 3=oman alueen sairaala)
Oman alueen sairaala (1 = HYKS, 2=Lappeenranta, 3=Lahti, 4=Kotka, jne)

PAD

Histologia (1=haiman dukt adenoca, 2=kolangioca, 3=IPMN, 4=NET,jne)
pT-luokka
pN-luokka
pM-luokka
Tutkittujen imusolmukkeiden määrä
Metastaattisten imusolmukkeiden määrä
R-luokka

Re-admissio, 30vrk (0 = ei, 1=kyllä)
Syy, vapaa kenttä

Paljastuiko randomisaatio hoitaville lääkäreille (0 = ei, 1 = kyllä, vahingossa, 2 = kyllä, sokkoutus purettiin tarkoituksellisesti)
Syy, vapaa kenttä

Randomisaatiolääkkeen lopetuspäivä, pvm

Randomisaatiolääkkeen mahdolliset haitat (0 = ei haittoja, 1 = pahoinvointi, 2 = kohonneet verensokerit, jne)

Jälkitarkastus kir pkl (0 = ei, 1=kyllä, HYKS)

Adjuvanttihoito (0 = ei, 1=gemsitabiini, 2=folfirinox, jnejne)
Adjuvanttihoidon aloituspäivä, pvm

Residivointi (0 = ei, 1=paikalllinen, 2=maksa, 3=keuhkot, 4=peritoneaalikarsinoosi, 5=multippeli)
Residivoinnin toteamispäivä, pvm

Kuollut (0 = ei, 1=kyllä, haimatuumorin aiheuttama, 2=kyllä, ei haimatuumoriin liittyvä, 3=kyllä,leikkauskomplikaatio)
Kuolinpäivä, pvm
Viimeinen seurantapäivä, pvm
Remissiossa (0 = ei, 1=kyllä)

Potilas halunnut keskeyttää tutkimuksen (0 = ei, 1=kyllä)
Keskeyttämispäivä, pvm
