# Supplementary material for: Effect of Hydrocortisone vs Pasireotide on Pancreatic Surgery Complications in Patients With High Risk of Pancreatic Fistula: A Randomized Clinical Trial
Source: JAMA Surg. 2020 Feb 5;155(4):291–8. doi: 10.1001/jamasurg.2019.6019 (PMC7042940; doi:10.1001/jamasurg.2019.6019)
Supplement: Supplement 2. — eTable 1. Cumulative Postoperative Complications Within 30 Days eTable 2. Primary and Secondary Outcomes for Patients Undergoing Pancreaticoduodenectomy eTable 3. Cumulative Postoperative Complications Within 30 Days in Pancreaticoduodenectomies eTable 4. Primary and Secondary Outcomes for Patients Undergoing Distal Pancreatectomy eTable 5. Cumulative Postoperative Complications Within 30 Days in Distal Pancreatectomies [file jamasurg-155-291-s002.pdf]

Supplementary Online Content

Tarvainen T, Sirén J, Kokkola A, Sallinen V. Effect of hydrocortisone vs pasireotide on pancreatic surgery complications in patients with high risk of pancreatic fistula: a randomized clinical trial. *JAMA Surg*. Published online February 5, 2020. doi:10.1001/jamasurg.2019.6019

eTable 1. Cumulative Postoperative Complications Within 30 Days

eTable 2. Primary and Secondary Outcomes for Patients Undergoing Pancreaticoduodenectomy

eTable 3. Cumulative Postoperative Complications Within 30 Days in Pancreaticoduodenectomies

eTable 4. Primary and Secondary Outcomes for Patients Undergoing Distal Pancreatectomy

eTable 5. Cumulative Postoperative Complications Within 30 Days in Distal Pancreatectomies

This supplementary material has been provided by the authors to give readers additional information about their work.

eTable 1. Cumulative postoperative complications within 30 days. Individual patients may have several complications reported.

Clavien-Dindo grade and type - no. (%)	Pasireotide (n = 63)	Hydrocortisone (n = 63)
None	***9 (14 %)***	***3 (5 %)***
1 (one or more per patient)	***45 (71 %)***	***54 (86 %)***
POPF	21 (33 %)	22 (35 %)
DGE	4 (6 %)	11 (18 %)
Chyloascites	9 (14 %)	6 (10 %)
Bile leak	0	2 (3 %)
Superficial wound infection (no need for antibiotics)	6 (10 %)	6 (10 %)
Electrolyte imbalance	17 (27 %)	37 (59 %)
Diuretics	26 (41 %)	22 (35 %)
Abnormally intensive post-operative pain	2 (3 %)	1 (2 %)
Pulmonary embolism[a]	0	1 (2 %)
Diarrhea	2 (3 %)	1 (2 %)
Neurological symptom	1 (1 %)	0
Spleen infarctation	1 (1 %)	0
2 (one or more per patient)	***39 (62 %)***	***35 (56 %)***
POPF	6 (10 %)	2 (3 %)
DGE	6 (11 %)	6 (10 %)
POPH	0	1 (2 %)
Bile leak	1 (2 %)	2 (3 %)
Chyloascites	1 (2 %)	0
Pneumonia	10 (16 %)	11 (18 %)
Pulmonary embolism	3 (5 %)	1 (3 %)
Congestive heart failure	1 (2 %)	1 (2 %)
Cholangitis	6 (10 %)	3 (5 %)
Deep abscess	4 (6 %)	3 (5 %)
Hypertonia	0	1 (2 %)
Urinary tract infection	1 (2 %)	2 (3 %)
Septicaemia	0	3 (5 %)
Arrythmia	2 (3 %)	3 (5 %)
Portal vein/SMV thrombosis	1 (2 %)	1 (2 %)
Hypoalbuminemia	9 (14 %)	11 (18 %)
Mesenterial embolism (arterial)	0	1 (2 %)
Neurological symptom	0	1 (2 %)
Paralytic ileus	0	1 (3 %)
Superficial wound infection (antibiotics needed)	0	1 (2 %)
Spleen infarctation	0	1 (2 %)
Deep vein thrombosis (limb)	0	1 (2 %)
Shortness of breath	0	1 (2 %)
3a (one or more per patient)	***11 (17 %)***	***21 (33 %)***
POPF	4 (6 %)	10 (16 %)
DGE	1 (2 %)	0
POPH	0	2 (3 %)
Pleural effusion, drainage	3 (5 %)	5 (8 %)
Post-operative pain, needing an epidural catether	1 (2 %)	0
Arrythmia, cardioversion	1 (2 %)	1 (2 %)
Liver ischemia, PTA	0	1 (2 %)
Deep abscesses, drainage	3 (5 %)	4 (6 %)
3b (one or more per patient)	***2 (3 %)***	***7 (11 %)***
POPF, laparotomy	1 (2 %)	2 (3 %)
DGE, laparotomy	0	1 (2 %)
POPH, laparotomy	0	4 (6 %)
Bile leak, laparotomy	0	2 (3 %)

Colon necrosis, laparotomy	1 (2 %)	0
Colonic anastomotic dehiscence, laparotomy	0	1 (2 %)[b]
4a (one or more per patient)	***2 (3 %)***	***2 (3 %)***
POPF, sepsis and relaparotomy	1 (2 %)	0
POPF, ERP stenting, severe post ERP pancretitis	0	1 (2 %)
Respiratory insufficiency	1 (2 %)	1 (2 %)
4b (one or more per patient)		
5 (death)	***1 (2 %)***	***2 (3 %)***
POPF	1 (2 %)	0
DGE leading to massive aspiration	0	1 (2 %)
Myocardial infarction leading to MOF and death	0	1 (2 %)

DGE – delayed gastric emptying, ERP – endoscopic retrograde pancreatography, MOF – multi-organ failure, POPF – postoperative pancreatic fistula, POPH – postoperative pancreatic haemorrhage, PTA – percutaneous transluminal angioplasty, SMV – superior mesenteric vein.

[a] Patient died on other causes and the treatment for embolism didn't get to be started hence C-D grade 1 for pulmonary embolism.

[b] Distal pancreatic tumor infiltrated colonic mesentery and a colon resection en-bloc with distal pancreatectomy with colonic anastomosis was performed

eTable 2. Primary and secondary outcomes for patients undergoing pancreaticoduodenectomy

	Pasireotide (N = 30)	Hydrocortisone (N = 27)	p value	Effect size OR (95% CI)
**Primary outcome**				
Mean Comprehensive Complication index (SD)	32·39 (17·83)	37·90 (17·56)	0·280	-5·51 (-15·46 – 4·12)[a]
**Secondary outcome**				
Complications, any CD)	30 (100 %)	30 (100 %)	1·000	-
Clinically significant complications (CD 2 or more)	24 (80%)	24 (89%)	0·476	2·00 (0·45 – 8·94)
Major complications (CD 3b or more)	5 (17 %)	4 (15%)	0·848	0·87 (0·21 – 3·64)
Pancreatic fistula, n (%)				
Any	21 (70%)	16 (59%)	0·396	0·623 (0·21 – 1·86)
ISGPS B/C	8 (27%)	7 (26%)	0·949	0·963 (0·30 – 3·14)
Delayed gastric emptying, n (%)				
Any	10 (33%)	15 (56%)	0·091	2·500 (0·86 – 7·31)
ISGPS B/C	5 (17%)	4 (15%)	1·000	0·870 (0·21 – 3·64)
Postoperative haemorrahge, n (%)				
Any	0	4 (15%)	**0·044**	N/A[b]
ISGPS B/C	0	4 (15%)	**0·044**	N/A[b]
Length of hospital stay, median (IQR)[c]	12·0 (8·0 – 17·0)	13·5 (10·0 – 19·0)	0·398	0·114[d]
Readmission, n (%)	3 (10%)[e]	3 (11%)	1·000	1·08 (0·20 – 5·89)
Adjuvant therapy among patients with cancer, n (%)[f]	11 (69%)	12 (80%)	0·685	1·82 (0·35 – 9·46)

CD – Clavien-Dindo, CI – confidence interval, ISGPS – International Study Group of Pancreatic Surgery, IQR – interquartile range, N/A – not applicable, OR – odds ratio, SD – standard deviation.

[a] Mean difference (95% CI)

[b] Effect size cannot be calculated due to a zero in one cell.

[c] One patient died during the initial hospital stay in both groups.

[d] Effect size was calculated as $r = Z/\sqrt{N}$ without 95% confidence interval.

[e] 1 patient's data missing about possible readmissions in his home town community hospital.

[f] Total N of cancer cases in Pasireotide group was 16 and in Hydrocortisone group 15. group Data of possible adjuvant therapy was missing in 4 patients in Pasireotide group and in 1 patient in Hydrocortisone due to their oncological consultation at other institution.

eTable 3. Cumulative postoperative complications within 30 days in pancreaticoduodenectomies. Individual patients may have several complications reported.

Clavien-Dindo grade and type - no. (%)	Pasireotide (Pancreatoduodenectomy) (n = 30)	Hydrocortisone (Pancreatoduodenectomy) (n = 27)
None	***0***	***0***
1 (one or more per patient)	***29 (97%)***	***26 (96%)***
POPF	13 (43%)	8 (30%)
DGE	3 (10%)	9 (33%)
Chyloascites	8 (27%)	4 (15%)
Bile leak	0	2 (7%)
Superficial wound infection (no need for antibiotics)	5 (17%)	3 (11%)
Electrolyte imbalance	14 (47%)	18 (67%)
Diuretics	20 (67%)	14 (52%)
Abnormally intensive post-operative pain	1 (3%)	1 (4%)
Pulmonary embolism[a]	0	1 (4%)
Diarrhea	1 (3%)	1 (4%)
Neurological symptom	1 (3%)	0
2 (one or more per patient)	***22 (73%)***	***21 (78%)***
POPF	3 (10%)	1 (4%)
DGE	6 (20%)	5 (19%)
POPH	0	1 (4%)
Bile leak	1 (3%)	2 (7%)
Chyloascites	1 (3%)	0
Pneumonia	3 (10%)	5 (19%)
Pulmonary embolism	2 (7%)	0
Cholangitis	5 (17%)	3 (11%)
Deep abscess	2 (7%)	2 (7%)
Urinary tract infection	0	1 (4%)
Septicaemia	0	3 (11%)
Arrythmia	1 (3%)	1 (4%)
Portal vein/SMV thrombosis	0	1 (4%)
Hypoalbuminemia	9 (30%)	8 (30%)
Mesenterial embolism (arterial)	0	1 (4%)
Neurological symptom	0	1 (4%)
Paralytic ileus	0	1 (4%)
Superficial wound infection (antibiotics needed)	0	1 (4%)
Deep vein thrombosis (limb)	0	1 (4%)
Shortness of breath	0	1 (4%)
3a (one or more per patient)	***6 (20%)***	***13 (48%)***
POPF	2 (7%)	5 (19%)
DGE	1 (3%)	0
POPH	0	1 (4%)
Pleural effusion, drainage	2 (7%)	4 (15%)
Arrythmia, cardioversion	0	1 (4%)
Liver ischemia, PTA	0	1 (4%)
Deep abscesses, drainage	3 (10%)	3 (11%)
3b (one or more per patient)	***2 (7%)***	***4 (15%)***
POPF, laparotomy	1 (3%)	1 (4%)
DGE, laparotomy	0	1 (4%)
POPH, laparotomy	0	2 (7%)
Bile leakage, laparotomy	0	2 (7%)
Colon necrosis, laparotomy	1 (3%)	0
4a (one or more per patient)	***2 (7%)***	***0***
POPF, sepsis and relaparotomy	1 (3%)	0

Respiratory insufficiency	1 (3%)	0
4b (one or more per patient)		
5 (death)	***1 (3%)***	***1 (4%)***
POPF	1 (3%)	0
DGE leading to massive aspiration	0	1 (4%)

[a] Patient died on other causes and the treatment for embolism didn't get to be started hence C-D grade 1 for pulmonary embolism.

eTable 4. Primary and secondary outcomes for patients undergoing distal pancreatectomy

	**Pasireotide (N = 30)**	**Hydrocortisone (N = 30)**	**p value**	**Effect size OR (95% CI)**
**Primary outcome**				
Mean Comprehensive Complication index (SD)	16·03 (11·94)	26·28 (21·76)	**0·033**	-10·25 (-19·34 to -2·12)[a]
**Secondary outcome**				
Complications, any CD	22 (73%)	28 (93%)	**0·038**	5·09 (0·98 – 26·43)
Clinically significant complications (CD 2 or more)	17 (57%)	18 (60%)	0·793	1·15 (0·41 – 3·20)
Major complications (CD 3b or more)	0	6 (20%)	**0·024**	N/A[b]
Pancreatic fistula, n (%)				
Any	11 (37%)	20 (67%)	**0·020**	3·46 (1·20 – 9·99)
ISGPS B/C	4 (13%)	6 (20%)	0·488	1·63 (0·41 – 6·47)
Delayed gastric emptying, n (%)				
Any	2 (7%)	3 (10%)	1·000	1·56 (0·24 – 10·05)
ISGPS B/C	1 (3%)	1 (3%)	1·000	1·00 (0·060 – 16·76)
Postoperative haemorrhage, n (%)				
Any	0	3 (10%)	0·237	N/A[b]
ISGPS B/C	0	2 (7%)	0·492	N/A[b]
Length of hospital stay, median (IQR)	7·0 (5·0 – 8·0)	7·0 (5·0 – 10·5)[c]	0·622	0·064[d]
Readmission, n (%)	4 (13%)	7 (23%)	0·317	1·98 (0·51 – 7·64)
Adjuvant therapy among patients with cancer, n (%)[e]	9 (90%)	5 (63%)	0·275	0·19 (0·02 – 2·29)

CD – Clavien-Dindo, CI – confidence interval, ISGPS – International Study Group of Pancreatic Surgery, IQR – interquartile range, N/A – not applicable, OR – odds ratio, SD – standard deviation.

[a] Mean difference (95% CI).

[b] Effect size cannot be calculated due to a zero in one cell.

[c] 1 patient died during the initial hospital stay.

[d] Effect size was calculated as $r = Z/\sqrt{N}$ without 95% confidence interval.

[e] Total N of cancer cases was 10 in Pasireotide group and 8 in Hydrocortisone group.

eTable 5. Cumulative postoperative complications within 30 days in distal pancreatectomies. Individual patients may have several complications reported.

Clavien-Dindo grade and type - no. (%)	Pasireotide (Distal pancreatectomy) (n = 30)	Pasireotide (Distal pancreatectomy) (n = 30)
None	***8 (13%)***	***2 (3%)***
1 (one or more per patient)	***15 (50%)***	***24 (80%)***
POPF	7 (23%)	12 (40%)
DGE	1 (3%)	2 (7%)
Chyloascites	1(3%)	2 (7%)
Superficial wound infection (no need for antibiotics)	1 (3%)	1 (3%)
Electrolyte imbalance	3 (10%)	17 (57%)
Diuretics	6 (20%)	7 (20%)
Abnormally intensive post-operative pain	1 (3%)	0
Diarrhea	1 (3%)	0
Spleen infarctation	1 (3%)	0
2 (one or more per patient)	***15 (50%)***	***12 (40%)***
POPF	2 (7%)	0
DGE	1 (3%)	1 (3%)
Pneumonia	7 (23%)	6 (20%)
Pulmonary embolism	1 (3%)	2 (7%)
Congestive heart failure	1 (3%)	1 (3%)
Cholangitis	1 (3%)	0
Deep abscess	2 (7%)	0
Hypertonia	0	1 (3%)
Urinary tract infection	1 (3%)	0
Arrythmia	0	2 (7%)
Portal vein/SMV thrombosis	1 (3%)	0
Hypoalbuminemia	0	3 (10%)
Paralytic ileus	0	1 (3%)
Spleen infarctation	0	1 (3%)
3a (one or more per patient)	***4 (13%)***	***8 (27%)***
POPF	2 (7%)	5 (17%)
POPH	0	1 (3%)
Pleural effusion, drainage	1 (3%)	1 (3%)
Arrythmia, cardioversion	1 (3%)	0
Deep abscesses, drainage	0	1 (3%)
3b (one or more per patient)	***0***	***3 (10%)***
POPF, laparotomy	0	1 (3%)
POPH, laparotomy	0	2 (7%)
Colonic anastomotic dehiscence, laparotomy	0	1 (3%)[a]
4a (one or more per patient)	***0***	***2 (7%)***
POPF, ERP stenting, severe post ERP pancretitis	0	1 (3%)
Respiratory insufficiency	0	1 (3%)
4b (one or more per patient)		
5 (death)	***0***	***1 (3%)***
Myocardial infarction leading to MOF and death	0	1 (3%)

[a] Distal pancreatic tumor infiltrated colonic mesentery and primarily a colon resection en-bloc with distal pancreatectomy with colonic anastomosis was performed.
