# Supplementary material for: Effect of Hydrocortisone vs Pasireotide on Pancreatic Surgery Complications in Patients With High Risk of Pancreatic Fistula: A Randomized Clinical Trial
Source: JAMA Surg. 2020 Feb 5;155(4):291–8. doi: 10.1001/jamasurg.2019.6019 (PMC7042940; doi:10.1001/jamasurg.2019.6019)
Supplement: Supplement 3. — Data Sharing Statement [file jamasurg-155-291-s003.pdf]

Data Sharing Statement

Tarvainen. Effect of Hydrocortisone vs Pasireotide on Pancreatic Surgery Complications in Patients With High Risk of Pancreatic Fistula. *JAMA Surg*. Published February 05, 2020.
10.1001/jamasurg.2019.6019

Data
Data available: No

Additional Information
Explanation for why data not available: The study permissions do not permit sharing of data outside research group.
